# Test−Retest Reliability of the ‘Welfare Quality^®^ Animal Welfare Assessment Protocol for Sows and Piglets’. Part 1. Assessment of the Welfare Principle of ‘Appropriate Behavior’

**DOI:** 10.3390/ani9070398

**Published:** 2019-06-29

**Authors:** Lena Friedrich, Joachim Krieter, Nicole Kemper, Irena Czycholl

**Affiliations:** 1 Institute of Animal Breeding and Husbandry, Christian-Albrechts-University, Olshausenstr. 40, 24098 Kiel, Germany; 2 Institute for Animal Hygiene, Animal Welfare and Farm Animal Behaviour, University of Veterinary Medicine Hannover, Foundation, Bischofsholer Damm 15, 30173 Hannover, Germany

**Keywords:** animal-based, animal welfare, behavior, pig, test−retest reliability, Welfare Quality^®^

## Abstract

**Simple Summary:**

The EU-funded Welfare Quality^®^ project generated species-specific assessment protocols to evaluate the welfare of pigs, poultry, and cattle. With the implementation to be used for certification purposes, it is important that the protocols show consistency over time, which describes the extent to which equal results are achieved if the assessment is performed repetitively. The protocols should not be sensitive to slight changes in the on-farm situation but mirror the long-term welfare situation on-farm. The present study aimed at testing this consistency over time of the indicators included in the ‘Welfare Quality^®^ animal welfare assessment protocol for sows and piglets’. Thereby, the study focused on the indicators to assess the welfare principle ‘appropriate behavior’. As a result, the indicators applied to assess the animals’ ‘appropriate behavior’ did not represent consistency over time. Thus, further investigation is needed before implementation on-farm. Conclusively, the present study contributes to the development of generally accepted and objective assessment protocols for animal welfare and thereby to the improvement of farm animals’ welfare overall.

**Abstract:**

The present study’s aim was to assess the test−retest reliability (TRR) of the ‘Welfare Quality^®^ animal welfare assessment protocol for sows and piglets’ focusing on the welfare principle ‘appropriate behavior’. TRR was calculated using Spearman’s rank correlation coefficient (RS), intraclass correlation coefficient (ICC), smallest detectable change (SDC), and limits of agreement (LoA). Principal component analysis (PCA) was used for deeper analysis of the Qualitative Behavior Assessment (QBA). The study was conducted on thirteen farms in Northern Germany, which were visited five times by the same observer. Farm visits 1 (F1; day 0) were compared to farm visits 2 to 5 (F2–F5). The QBA indicated no TRR when applying the statistical parameters introduced above (e.g., ‘playful‘ (F1–F4) RS 0.08 ICC 0.00 SDC 0.50 LoA [−0.62, 0.38]). The PCA detected contradictory TRR. Acceptable TRR could be found for parts of the instantaneous scan sampling (e.g., negative social behavior (F1–F3) RS 0.45 ICC 0.37 SDC 0.02 LoA [−0.03, 0.02]). The human−animal relationship test solely achieved poor TRR, whereas scans for stereotypies showed sufficient TRR (e.g., floor licking (F1–F4) RS 0.63 ICC 0.52 SDC 0.05 LoA [−0.08, 0.04]). Concluding, the principle ‘appropriate behavior’ does not represent TRR and further investigation is needed before implementation on-farm.

## 1. Introduction

Relating to the five freedoms of the Farm Animal Welfare Council (FAWC), animal welfare is a concept with multiple dimensions, which consists of the absence of thirst, hunger, discomfort, pain and injuries, stress, and the expression of normal behavior [1]. Thus, animal welfare involves not only physical but also mental health [2,3]. With increasing public and political concern for animal welfare [4], the demand for assessment protocols of general acceptance and objectivity to assess the welfare of farm animals has also increased [5]. The generation of such broadly accepted and objective assessment protocols for animal welfare was the aim of the EU-funded Welfare Quality^®^ project. To account for the multidimensional base of animal welfare, the Welfare Quality^®^ protocols were developed using a multicriteria approach: four main principles were identified for the assessment of animal welfare—‘good feeding’, ‘good housing’, ‘good health’, and ‘appropriate behavior’ [6].

A measurement method is characterized as objective when it is feasible, valid, and reliable [7]. Feasibility refers to the practical implementation of the method (i.e., that the execution produces reliable results at acceptable costs) [7]. An indicator is called valid when it achieves true results measuring what it is assumed to measure [8]. Reliability describes the analogy between repetitive measurements of the same object (i.e., the repeatability and consistency of an indicator). One subsection of reliability is the test−retest reliability. This represents the consistency of a method over time, consequently, the extent to which equal results are achieved if the assessment is performed repetitively [8,9]. Consistency over time is particularly important if the method is intended to be used for certification because the certification process cannot be repeated frequently for reasons of time and cost-effectiveness [10]. The Welfare Quality^®^ protocols are optimal when they accurately predict outcomes over a long period of time (i.e., at least six months) [11]. Thus, the assessments should not detect inevitable, slight changes in the on-farm situation [12]. Consequently, assessment methods with good test−retest reliability would achieve the same outcomes even in the presence of slight changes in the on-farm situation that occur over time [13].

The indicators included in the Welfare Quality^®^ protocols were chosen with regard to feasibility, validity, and reliability. However, protocols in their entirety, have been rarely tested, in particular the Welfare Quality^®^ protocol for sows and piglets. Available assessments of reliability for the Welfare Quality^®^ protocols for sows and piglets have previously relied on video sequences collected and applied under experimental conditions to reduce time and cost rather than being conducted on-farm [14]. Videos provide a standardized assessment situation, which is barely influenced by the environment [15]. Thus, the on-farm testing is important to compromise all influences on reliability and mirror the actual assessment situation like for instance influenced by lighting in the stable.

As a consequence, the present study represents the first time the ‘Welfare Quality^®^ animal welfare assessment protocol for sows and piglets’ underwent on-farm testing for reliability on breeding to wean production animals. This paper focusses on the evaluation of the test−retest reliability of the welfare principle of ‘appropriate behavior’ as described in the protocol. The results on the remaining principles (‘good feeding’, ‘good housing’, and ‘good health’) are published in Friedrich et al. [16]. Consequently, the present study contributes to the development of an objective assessment system for animal welfare in sows and piglets, which may be used for certification purposes.

## 2. Materials and Methods

### 2.1. Data Collection

All data collection was performed by a single, trained observer. Training was provided by official members of the Welfare Quality^®^ consortium. Furthermore, a preliminary study with ten farm visits was performed to guarantee good training status before the main data collection started. The results presented do not include the data of this preliminary study.

Data collection took place on thirteen farms in Schleswig−Holstein, Germany between September 2016 and April 2018. The farms participated on a voluntary basis and varied among others in their production type (conventional or organic), herd size (40 to 5000 sows), and production rhythm (one-week to four-week rhythm). Selected characteristics of the farms are given in Table 1.

Each farm was visited five times over a period of ten months in total per farm with the same assessment intervals on each farm (day 0, day 3, week 7, month 5, month 10). The assessment intervals chosen for this study, especially the intervals of five and ten months, are in line with the intervals advised in literature to comply with feasibility and cost-effectiveness [11]. The time between farm visits on different farms was nine days on average ranging from two days to 49 days, which guaranteed continuous practice of the observer. The complete Welfare Quality^®^ protocol for sows and piglets was performed during each visit. However, the present paper focuses on the evaluation of the assessment of the welfare principle of ‘appropriate behavior’.

### 2.2. Protocol Assessment

This paper focuses on the methods included in the Welfare Quality^®^ protocol for sows and piglets applied for the assessment of the welfare principle of ‘appropriate behavior’. The methods comprise a Qualitative Behavior Assessment (QBA), behavioral observations by instantaneous scan sampling to assess social and exploratory behavior (ISS), a human−animal relationship test (HAR) and the assessment of stereotypies (ST). The methods are described in more detail in the following sections, with a focus on the characteristics of the present study. The specifications of the protocol were strictly followed during the study. Further information describing the methodology of the Welfare Quality^®^ protocol is available in [17].

#### 2.2.1. Qualitative Behavior Assessment (QBA)

The QBA is designed to assess animals’ expressive quality of behavior or ‘body language’ (i.e., it describes animal behavior, interaction with other animals and the environment) [17]. The QBA was performed at four to six observation points, whereby the number of observation points usually depended on the size and structure of the farm. The objective was to observe the expressive quality of behavior in farrowing, breeding, and gestation unit on each farm. The total observation time was 20 min per farm, which had to be divided depending on the number of observation points. In the given observation time, the observer watched the expressive quality of the activities of all the animals that could be seen clearly from each observation point. Afterwards, the ‘body language’ of the animals was assessed by summarizing the animals of all observation points in one score. The assessment included the evaluation of 20 adjectives (1: active; 2: relaxed; 3: fearful; 4: agitated; 5: calm; 6: content; 7: tense; 8: enjoying; 9: frustrated; 10: sociable; 11: bored; 12: playful; 13: positively occupied; 14: listless; 15: lively; 16: indifferent; 17: irritable; 18: aimless; 19: happy; 20: distressed). Each of the 20 adjectives was rated on a visual analogue scale of 125 mm from absent (0 mm) to dominant (125 mm). In doing so, the label ‘absent’ indicated that the expressive quality of the evaluated adjective was totally missing in all observed animals. The opposite side of the scale indicated that the adjective was predominant in the observed animals.

#### 2.2.2. Behavioral Observations by Instantaneous Scan Sampling (ISS)

An ISS is prescribed in the protocol to evaluate social behavior, exploratory behavior, and other active behavior in gestating sows. The ISS took place in the morning when the animals were more active and avoided the feeding period if animals were not fed ad libitum. The number of locations depended on the group size (small groups (<15 sows): four pens, intermediate groups (15–40 sows): two pens, large groups (>40 sows): one pen). The pens on farms with small groups were chosen to be evenly distributed (i.e., pens on each side and in the middle of the gestation unit were included) to generate an overall picture of the animals. Before starting the ISS, the animals in the pens chosen for the observations were roused. This was done by walking around in the chosen pen. A calming down time of 5 min was applied from outside the pen. Subsequently, the ISS was carried out from outside the pen as well for the total observation time of 10 min using five scan samples at intervals of 2 min. The pigs were either evaluated as active or as inactive. The evaluation of the active pigs included the number of animals performing positive and negative social behavior, exploring enrichment material and performing an investigation of the pen, and other active behaviors such as drinking or walking.

#### 2.2.3. Human−Animal Relationship Test (HAR)

The HAR was performed after the ISS because the sows were already aware of the observer. The HAR comprised a sample of 20 sows in the gestation unit. The sows were selected complying with the sampling described by the protocol. Thus, all animals were assessed in small groups (<6 sows). A representative number out of each pen was involved when having intermediate groups (≥6 sows). It was attempted in both small and intermediate groups to evenly distribute the pens across the gestation unit as in the ISS. A random selection was applied in large groups (≥100 sows). Therefore, the first sow in the pen was set as the ‘starting sow’. After performing the HAR with the ‘starting sows’, the observer moved on towards the fourth sow looking from the direction of the ‘starting sow’. This process was repeated until 20 sows had been assessed. The HAR consisted of three stages: first, the observer stood in front of the sow for 10 s. If the sow did not react, the observer proceeded to stage 2 and crouched down in front of the sow and stayed motionless again for 10 s. If the sow did not react, the observer proceeded to stage 3, where the observer tried to touch the sow between the ears. The HAR was scored on a three-point scale (0 = no fear response, i.e., the sow permitted to be touched between the ears (stage 3), 1 = light fear response, i.e., the sow refused to be touched (stage 3), 2 = strong fear response, i.e., the sow withdrew when the observer stood or crouched down in front of her (stage 1 or stage 2)).

#### 2.2.4. Stereotypies (ST)

A random sample of 40 sows in the gestation unit were observed for stereotypical behavior including the ST behaviors sham chewing, tongue rolling, teeth grinding, bar, drinker, trough biting and floor licking. The sampling process was carried out as explained in the HAR. The ST assessments took place in the morning when the sows were more active and avoided the feeding period. The total observation time per sow was 15 s. The observation period could be extended up to 30 s if the observer was unsure whether the sow was performing stereotypical behavior. A binary score (0 = absent, 2 = present) was applied for the assessment of ST.

### 2.3. Statistical Analysis

The results of the QBA were calculated for each adjective by reading out the length (mm) on the visual analogue scale with a ruler. Subsequently, the scores in mm were divided over the total length of the visual analogue scale. Thus, the dataset contained one percentage score for each adjective and each farm visit to every farm (e.g., farm 1, farm visit 1: ‘happy’ 52%; range for each adjective: 0–100%). The sum of the 20 adjectives in one farm visit did not account for 100% because adjectives were not mutually exclusive and therefore one animal could be rated as more than one adjective at the same time. The results of the ISS were expressed as a percentage of the total active behavior during each farm visit to every farm (e.g., farm 1, farm visit 1: positive social behavior: 5%, negative social behavior: 4%, use of enrichment material: 10%, investigation of the pen: 6%, other active behavior: 75%; sum: 100%). Finally, the percentage of animals within each category of the HAR and ST was calculated for each farm visit to every farm (e.g., farm 1, farm visits 1: HAR category 0: 90%; HAR category 1: 6%; HAR category 2: 4%; sum = 100%). The results of QBA, ISS, HAR, and ST represent a random sample of the population within a farm and provide an overview of farm level dynamics. Consequently, for each of the five farm visits, the dataset contained thirteen observations, which was equivalent to the number of farms included in the present study. A pairwise comparison was carried out between farm visits 1 (F1; day 0) as a reference and subsequent farm visits (F2–F5; day 3, week 7, month 5, month 10) for the adjectives of the QBA, the categories of the ISS, and each category of the HAR and ST. Therefore, Spearman’s rank correlation coefficient (RS) and intraclass correlation coefficient (ICC) were calculated as reliability parameters, whereas smallest detectable change (SDC) and limits of agreement (LoA) were calculated as agreement parameters. All statistical analyses were performed using the statistical software SAS^®^ 9.4. [18]. The RS was calculated by the procedure PROC CORR. The procedure PROC GLM was applied to calculate the ICC. The SDC, as derived from the ICC, and LoA were calculated using the formulas which are explained below. The QBA was further analyzed by means of principal component analysis (PCA). The statistics are described in detail in the following.

#### 2.3.1. Spearman’s Rank Correlation Coefficient (RS)

The RS is a non-parametric measure of rank correlation [19]. Rank correlations range from −1.00 to 1.00, with positive correlations closer to 1.00 providing greater confidence for test−retest reliability. The RS is calculated by:

(1) $$\mathrm{RS}\;=\;\frac{1\;-\;6\;\sum_{i=1}^{n}d_{i}^{2}}{n^{3\;}-\;n},$$

with d_i_ being the difference between the ranks for each x_iyi_ data pair, and n being the number of data pairs [19]. In the present study, using guidance from Martin and Bateson [8], RS equal to or greater than 0.40 was interpreted as acceptable reliability and RS equal to or greater than 0.70 was interpreted as good reliability.

#### 2.3.2. Intraclass Correlation Coefficient (ICC)

Variance is the basis of the ICC. Thereby, the ICC places the variance between study objects (farms) in proportion to the variance between study objects plus measurement error [20]. For the analysis of variance, the following two-way model regarding to Shrout and Fleiss [21] and McGraw and Wong [22] was assigned:

X_ij_ = µ + α_i_ + β_j_ + ε_i,j_, (2)

with X_ij_ being the measured value, µ the general average value, α_i_ the random effect of the differences between the study objects (farms), β_j_ the fixed effect of the farm visits, and ε_ij_ as the general error term.

The ICC was calculated with regard to the formula of consistency, which was published by de Vet et al. [20], in the following way:

(3) $$\mathrm{ICC}\;=\;\frac{\sigma^{2}\;(\mathrm{objects})}{\sigma^{2}\;\left(\mathrm{objects}\right)\;+\;\sigma^{2}\;(\mathrm{residual})\;},$$

with σ^2^ representing the variance of the study objects (farms) and the residual variance, respectively.

By definition, ICC can range between 0.00 and 1.00, whereby a value of 0.00 indicates the total absence of reliability and a value of 1.00 indicates perfect reliability. Regarding the interpretation, an ICC equal to or greater than 0.40 implied acceptable reliability and an ICC equal to or greater than 0.70 implied good reliability [22].

#### 2.3.3. Smallest Detectable Change (SDC)

The SDC is an expression of the measurement error, which contains the residual variance. According to de Vet et al. [20], the SDC is calculated by

SDC = 1.96 × (√2 × σ^2^_(residual)_), (4)

with σ^2^ being the residual variance.

The SDC outputs the smallest change in the score that can be detected despite the measurement error. Thereby, the values of SDC correspond to the measurement unit of the indicators under assessment. In the present study, the measurement unit was displayed in percent as a decimal number. Relating to the simple agreement coefficient calculated by de Vet et al. [20], a SDC smaller than or equal to 0.10 indicated acceptable agreement, a SDC smaller than or equal to 0.05 good agreement.

#### 2.3.4. Limits of Agreement (LoA)

The LoA were calculated with regard to the formula named below, which corresponds to de Vet et al. [20]:

LoA = mean ± 1.96 × (√2 × σ^2^_(residual)_), (5)

with σ^2^ representing the residual variance.

The LoA estimates the differences between two sets of measurement values. In this case, these were the differences of the measurements obtained between the farm visits and the standard deviation of these differences. Most of the differences are expected to be less than two standard deviations. In this study, LoA is expressed as a relative frequency between −1.00 and 1.00. Again, the interpretation was based on the simple agreement coefficient of de Vet et al. [20] and therefore an interval smaller than or equal to −0.10 to 0.10 was interpreted as acceptable agreement, an interval smaller than or equal to −0.05 to 0.05 as good agreement.

#### 2.3.5. Final Evaluation of Test−Retest Reliability

For better understanding, the term ‘reliability’ is used throughout the manuscript when referring to the results of the reliability parameters RS and ICC, the term ‘agreement’ is applied for the results of the agreement parameters SDC and LoA. The differentiation between reliability and agreement parameters and their interpretation are further discussed in the section ‘Reliability and agreement parameters’. The final evaluation, which summarizes all statistical parameters, is covered by the term ‘test−retest reliability’.

According to the definitions introduced above, an acceptable test−retest reliability was obtained when RS and ICC were equal to or greater than 0.40, when SDC was equal to or smaller than 0.10, and when LoA was equal to or smaller than −0.10 to 0.10. Ideally, an indicator achieved acceptable test−retest reliability when all statistical parameters reached the thresholds for acceptability. However, two exceptional cases were defined: On the one hand, the test−retest reliability was rated as acceptable when the repeated farm visits were close to each other, which is indicated by acceptable agreement in the statistical parameters concerning agreement (SDC equal to or smaller than 0.10 and LoA equal to or smaller than −0.10 to 0.10). On the other hand, the test−retest reliability was evaluated as acceptable when the farms could be distinguished from each other within the repeated farm visits, which was indicated by acceptable reliability in the statistical parameters in relation to reliability (RS and ICC equal to or greater than 0.40).

#### 2.3.6. Principal Component Analysis (PCA)

The PCA was performed for further analysis of the QBA as advised by Wemelsfelder et al. [23,24]. Therefore, the procedure PROC FACTOR was applied. Following Temple et al. [10], raw data were transformed into a correlation matrix. For the analysis, a single PCA was calculated for each farm visit (F1–F5). In doing so, no rotation was applied. The first two principal components (PC; PC1 and PC2), which had an eigenvalue of greater than 1.00, were used for the comparison. Each adjective achieved a certain factor loading on PC1 and PC2, which is a dimensionless number between −1.00 and 1.00. Finally, the factor loadings on PC1 and the factor loadings on PC2 were compared between the farm visits (F1 vs. F2, F1 vs. F3, F1 vs. F4, F1 vs. F5) by means of RS. Thereby, F1 counted as a reference value as in the previous analyses. A RS equal to or greater than 0.40 was evaluated as acceptable correlation and a RS equal to or greater than 0.70 as good correlation [8]. Further, the RS was used to determine the correlation between the adjectives of the QBA. A correlation matrix based on RS was used to sort the adjectives of the QBA into different groups. The underlying hypothesis was that the expressive quality of behavior can be subdivided into distinct groups, which may achieve varying degrees of test−retest reliability. Subdivided into groups, the PCs, which were calculated as explained above, were compared for each group of adjectives.

### 2.4. Ethical Statement

The authors declare that the experiments were carried out strictly following international animal welfare guidelines. The animals on the farms were housed conventionally or according to the EU organic scheme [25]. In both cases, the animals were kept according to EU and national law (‘German Animal Welfare Act’ (German designation: TierSchG) [26] and the ‘German Order for the Protection of Production Animals used for Farming Purposes and other Animals kept for the Production of Animal Products’ (German designation: TierSchNutztV) [27]). Further, the animals were handled according to the ‘German Order for the Protection of Animals used for Experimental Purposes and other Scientific Purposes’ (German designation: TierSchVersV) [28]. No pain, suffering or injury was inflicted on the animals during the experiments.

## 3. Results

### 3.1. Qualitative Behavior Assessment (QBA)

Table 2 contains the mean values in percent and the standard error obtained for the different farm visits. Table 3 presents the corresponding statistical parameters regarding the test−retest reliability for the adjectives of the QBA.

No test−retest reliability could be found in the comparison of the percentages of any of the adjectives. Even though some adjectives indicated reliability in terms of the RS and ICC reliability parameters for some farm visits, e.g., the adjectives ‘agitated’, ‘content’, ‘enjoying’, ‘lively’, and ‘happy’, the reliability was not consistent across all farm visits and the agreement indicated by the agreement parameters SDC and LoA was low in all adjectives and for all farm visits.

**Figure 1:**
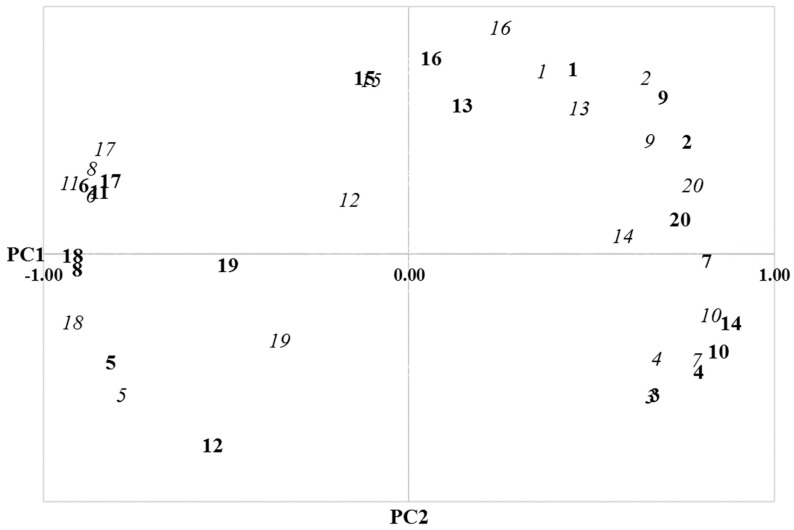
Plotted factor loadings of the first two principal components (PC; PC1 and PC2) for the comparison of farm visit 1 (bold type) and farm visit 3 (italic type) (adjectives: active = 1, agitated = 2, aimless = 3, bored = 4, calm = 5, content = 6, distressed = 7, enjoying = 8, fearful = 9, frustrated = 10, happy = 11, indifferent = 12, irritable, 13, listless = 14, lively =15, playful = 16, positively occupied = 17, relaxed = 18, sociable = 19, tense = 20).

The QBA was further analyzed by means of PCA. The factor loadings on the first two components explained 75.6% of the variance in F1, 68.7% of the variance in F2, 69.0% of the variance in F3, 67.3% of the variance in F4, and 70.3% of the variance in F5. Proceeding, the factor loadings were plotted in a two-dimensional interpretative word chart, which is shown as an example for the comparison of F1 with F3 in Figure 1.

The test−retest reliability of the PCA detected similar results as the direct comparison of the terms of the QBA. The values achieved by RS are presented in Table 4.

Table 4 indicates that PCA detected no test−retest reliability between F1 and F2. In contrast, the results demonstrated good test−retest reliability for the comparison of F1 with F3. Insufficient test−retest reliability was achieved between F1 and F4 and between F1 and F5.

With regard to the correlations among the adjectives in all farm visits for all farms, the adjectives of the QBA could be sorted into the following groups: group 1 (RS: 0.63–0.92): ‘relaxed’, ‘happy’, ‘enjoying’, ‘content’, ‘positively occupied’, ‘sociable’; group 2 (RS: 0.48–0.79): ‘lively’, ‘playful’, ‘irritable’, ‘active’, ‘fearful’, ‘agitated’, ‘tense’; group 3 (RS: 0.42–0.82): ‘aimless’, ‘bored’, ‘frustrated’, ‘distressed’, ‘listless’, ‘calm’, ‘indifferent’.

The factor loadings on PC1 and PC2 were compared subdivided by the groups introduced above. The values of RS are shown in Table 5.

It becomes visible that group 3 achieved an overall poor test−retest reliability. An acceptable test−retest reliability could be detected for the comparison of F1 and F4 in group 1 as well as for the comparisons of F1 and F3 and F1 and F4 in group 2. However, none of the groups obtained acceptable test−retest reliability in all comparisons.

### 3.2. Behavioral Observations by Instantaneous Scan Sampling (ISS)

Table 6 shows the mean values in percent and the standard error obtained for the different farm visits. Table 7 displays the corresponding statistical parameters concerning the test−retest reliability for the categories of the ISS.

Relating the sorting of the percentages of animals in the categories of the ISS, discrepancies could be detected between farm visits. In terms of reliability, the reliability parameters RS and ICC detected at large low reliability for the categories ‘positive social behavior’, ‘negative social behavior’, and ‘investigation of the pen’. The categories ‘use of enrichment material’ and ‘other active behavior’ achieved reliability in the comparisons of F1 and F2 to F4 but lacked both for reliability in the comparison of F1 and F5. In terms of agreement, expressed by the statistical parameters SDC and LoA, poor agreement was achieved by the categories ‘use of enrichment material’ and ‘other active behavior’, while the categories ‘positive social behavior’, ‘negative social behavior’, and ‘investigation of the pen’ showed acceptable to good agreement across all comparisons.

### 3.3. Human−Animal Relationship Test (HAR)

In the assessment of the HAR, the reliability parameters RS and ICC detected acceptable reliability for category 0 across all comparisons. In contrast, the reliability parameters for categories 1 and 2 were not consistent over time throughout data collection. Further, no agreement was obtained in the agreement parameters SDC and LoA in any of the categories.

Table 8 presents the mean values in percent and the standard error obtained for the different farm visits for the assessment of the HAR and ST. Table 9 contains the corresponding statistical parameters concerning the test−retest reliability.

### 3.4. Stereotypies (ST)

The results of the assessment of ST are presented in Table 8 and Table 9 as described above. The indicators sham chewing and tongue rolling showed acceptable to good reliability in terms of the reliability parameters RS and ICC. Relating to the agreement parameters SDC and LoA, only poor agreement could be found. The indicators teeth grinding, bar, drinker, trough biting, and floor licking achieved poor reliability regarding the reliability parameters RS and ICC but overall good agreement in terms of the agreement parameters SDC and LoA.

## 4. Discussion

### 4.1. Reliability and Agreement Parameters

For the evaluation of reliability, the use of a variety of reliability and agreement parameters is advised by de Vet et al. [20] to strengthen the advantages of the parameters and to compensate for possible disadvantages as the interpretation of only one parameter can end up in misinterpretation. The statistical parameters used in the present study were chosen with regard to welfare-related reliability studies which have been performed by Czycholl et al. [29,30] and Temple et al. [10]. In terms of the comparability of the studies, it is suitable to use the same statistical parameters. 

The RS and the ICC are correlation coefficients and measurements of reliability and thus they indicate the degree of differentiation between study objects despite the measurement error. The ICC is limited by its strong dependency on the total variance of the objects under assessment. This dependency on the total variance needs to be borne in mind while analyzing reliability [31]. Hence, if the study objects vary widely, the ICC achieves greater values, but it becomes very small despite good reliability if the study objects do not vary much from each other [20]. The RS is influenced by the presence of ties. It is underestimated if many ties exist within the data [32].

Statistical parameters of agreement are SDC and LoA—even though the SDC is mathematically derived from the ICC. The SDC and the LoA assess of how close results of repetitive measurements are by estimating the measurement error [20,33]. As an advantage, these parameters do not depend on the variance of the data. However, the subjective definition of threshold values remains somewhat difficult. For this reason, the threshold values of the present study were oriented towards existing reliability studies [10,16,29,30] and defined as advised by de Vet et al. [20].

The analysis of the test−retest reliability within the present study was performed at farm level to generate an overall picture of the farms because the animals were chosen to be a random sample of the whole population of the farms. The data for the QBA and the ISS was already available at farm level because the assessment was performed in groups of sows. In contrast, the animals, which were assessed at individual animal level in the HAR and ST, had to be sorted into the corresponding categories of the HAR and ST to prepare the data for analysis as explained above. The categories of the ISS, HAR, and ST were analyzed individually and independently. While the categories are not independent as each animal was placed in a single category, the chosen approach was used to reveal differences between defined categories and to ensure comparability with studies on the test−retest reliability of the Welfare Quality^®^ protocol for growing pigs performed by Temple et al. [10] and by Czycholl et al. [29] and on the test−retest reliability of the Welfare Quality^®^ protocol for sows and piglets performed by Friedrich et al. [16].

Reliability, together with feasibility and validity, characterize an objective measurement method [7]. Thereby, especially the test−retest reliability and the accompanying consistency over time are important characteristics for methods which might be used as certification tools of farms [10]. In terms of the interpretation, there are three causes for low test−retest reliability according to Temple et al. [10]: First, true differences between the farm visits exist. This factor was minimized as far as possible as no planned changes concerning the management of the farms occurred while the data were being collected. Second, differences in the assessments were detected due to the sensitivity of the measurement method and were caused by minor changes between the farm visits. According to Knierim and Winckler [11], feasible welfare assessment tools have to be carried out at longer time intervals of greater than six months to be useful for certification purposes. This is why the methods should not be sensitive to minor changes [10]. For instance, the outcome of a certification should not depend on the weekday of assessment. Third, low reliability can result of methodological restrictions (i.e., the exact reason cannot be specified, but nevertheless, the indicator in its present form is not suitable for the assessment).

### 4.2. Qualitative Behavior Assessment (QBA)

The direct comparison of the percentages of each adjective of the QBA within the five farm visits showed no test−retest reliability. In no case did all statistical parameters (RS, ICC, SDC, LoA) indicate at least acceptable test−retest reliability. The reliability parameters RS and ICC achieved good reliability for some adjectives in some farm visits, but their values varied constantly over the time. Thus, the conclusion cannot be drawn that at least a correlation between the farm visits existed. The agreement parameters SDC and LoA indicated poor agreement among all farm visits and for all adjectives. The results correspond to findings by Czycholl et al. [30], who tested the test−retest reliability of the ‘Welfare Quality^®^ animal welfare assessment protocol for growing pigs’.

The PCA is a widely used measurement tool which analyzes the results of the QBA in relation to redundancies between the adjectives [23,24,34]. The results of the PCA indicated low test−retest reliability because the RS values varied widely. No test−retest reliability could be detected between F1 and F2. In contrast, good test−retest reliability was achieved between F1 and F3. Overall, insufficient test−retest reliability was found between F1 and F4 and between F1 and F5.

The results of the present study correspond to studies performed in growing pigs. On-farm studies on growing pigs [10,35] detected all-embracing, low test−retest reliability using PCA as an analysis tool for the QBA. In contrast, Phythian et al. [36] detected a high consistency of the QBA over time in an on-farm study on sheep.

On the one hand, the QBA may have been inconsistent over time due to true variations in the physical and mental state of the animals [10]. If this is the case, the method is too sensitive and reacts to minor changes because no major changes (e.g., change of breed or production rhythm) were applied within data collection. As explained above, assessment systems, which are considered to be used for certification, should detect the long-term welfare situation on a farm and should not react to (rather meaningless) changes [10,11]. Consequently, the QBA shows poor test−retest reliability and is therefore not appropriate for certification purposes in terms of animal welfare assessment.

On the other hand, the results may have been affected by an intraobserver effect. For instance, Dalmau et al. [37] discussed overwork of observers when having to remember multiple assessment like the assessments of each observation point in the final evaluation of the QBA. Thus, adjustments to the execution of the QBA—like for example independent QBA for each observation point—may positively contribute to the reliability of the QBA. However, it is not possible to differentiate between true changes in the animals and a low intraobserver reliability [10]. Thus, further studies are needed to verify the results of the present study.

Further, low variance between the farms may have resulted in a weaker correlation in the QBA [10,35]. According to Phythian et al. [36], sheep farms vary widely regarding their husbandry and housing conditions, and thus provide a large internal variance in general, which has a positive effect on the reliability in the QBA. The present study included farms in the data collection which varied with regard to their production type (conventional or organic), herd size (40 to 5000 reproductive sows), and production rhythm (one-week to four-week rhythm) to generate a high variance. Still, being restricted to farms in Northern Germany and having no definition for low or high variance, the present study should be considered as a case study and future studies are needed to finally evaluate the QBA.

The correlation between the adjectives of the QBA formed three groups. With regard to the adjectives included in each groups, they were interpreted as group 1 corresponding to ‘positive emotions’ (‘relaxed’, ‘happy’, ‘enjoying’, ‘content’, ‘positively occupied’, ‘sociable’), group 2 corresponding to ‘active behavior’ (‘lively’, ‘playful’, ‘irritable’, ‘active’, ‘fearful’, ‘agitated’, ‘tense’), and group 3 corresponding to ‘negative emotions’ (‘aimless’, ‘bored’, ‘frustrated’, ‘distressed’, ‘listless’, ‘calm’, ‘indifferent’).

The group involving ‘active behavior’ achieved good test−retest reliability for the comparisons of F1 and F3 and F1 and F4, which was the best result among all groups. Nevertheless, neither the group of ‘active behavior’ nor one of the other groups obtained at least acceptable test−retest reliability across all farm visits. Concerning the group of ‘active behavior’, activity is likely to be scored more easily because changes in the activity are already a common method to assess animal well-being [38]. Further, the assessment of ‘active behavior’ may be more objective and therefore may be easier to perform because—in contrast to emotions—‘active behavior’ can be clearly defined [39]. However, Meagher et al. [39] raised the concern that the adjectives of the QBA may not be suitable for application in research at all. Bearing in mind the need for explicit definitions, they outlined that no definite definitions for adjectives, such as for example ‘distressed’, are obtainable. Consequently, in terms of their test−retest reliability, none of the adjectives used in the QBA can be recommended to assess the animals’ expressive quality of behavior—even if the group of ‘active behavior’ presented consistency over time in some comparisons.

In closing, it is not possible to differentiate whether the animals’ emotional state was really inconsistent over time or whether intraobserver reliability or inter-farm variability may have influenced the results of the test−retest reliability of the QBA. Therefore, future studies are needed to verify the result of poor test−retest reliability for the QBA found in the present study. Nevertheless, compliance with other studies on growing pigs supports the credibility of the present results. Further, the present study attempted to prevent complications due to low variance by including different farms, which was at least sufficient to minimize the influence of low inter-farm variability.

### 4.3. Behavioral Observations by Instantaneous Scan Sampling (ISS)

In total, the ISS showed acceptable test−retest reliability for the categories ‘positive social behavior’, ‘negative social behavior’, and ‘investigation of the pen’. Only poor test−retest reliability was achieved by the categories ‘use of enrichment material’ and ‘other active behavior’. 

The categories ‘positive social behavior’, ‘negative social behavior’, and ‘investigation of the pen’ achieved low reliability, which was pointed out by the values of the reliability parameters RS and ICC, whereas good agreement was pointed out by the agreement parameters SDC and LoA. The behavioral categories ‘positive social behavior’, ‘negative social behavior’, and ‘investigation of the pen’ appeared only with low prevalences, which possibly could be a reason for the low reliability of these categories despite good agreement in the agreement parameters SDC and LoA. The RS is influenced by existing ties, whereas the ICC depends on the variance. Thus, while having more ties in the data, RS becomes under-estimated [32]. While having close values of the study objects, the measurement error influences the capacity to distinguish the study objects and the reliability becomes lower in the ICC [20]. Agreement parameters on the other hand evaluate the extent to which the same value was assigned to an object within the different farm visits. Thereby, agreement parameters are not affected by the variance of the data. Consequently, Wirtz and Caspar [31] recommended that the test−retest reliability of an indicator should be rated as acceptable—even though with low values in RS and ICC—when the agreement parameters report the equality of values between the study objects. Therefore, the categories ‘positive social behavior’, ‘negative social behavior’, and ‘investigation of the pen’ can be rated as acceptable concerning their test−retest reliability.

In contrast, the categories ‘use of enrichment material’ and ‘other active behavior’ achieved poor reliability in terms of RS and ICC and poor agreement with regard to SDC and LoA. Thus, representing poor reliability and poor agreement, the test−retest reliability can only be evaluated as poor in total.

The results of the present study are in line with findings by Czycholl et al. [30] concerning growing pigs. Czycholl et al. [30] detected good test−retest reliability in the categories ‘positive social behavior’ and ‘negative social behavior’ using the agreement parameters SDC and LoA. They explained their worse agreement in the category ‘investigation of the pen’ by difficulties in the differentiation between this category and the category ‘other active behavior’ because both types of behavior may change very rapidly. This may also be an explanation for the poor test−retest reliability in the categories ‘use of enrichment material’ and ‘other active behavior’ of the present study.

Further, the practical experience raised the question of whether the renovation of enrichment material may have caused a bias between the categories ‘use of enrichment material’ and ‘other active behavior’ with more sows performing ‘use of enrichment material’ when provided new enrichment material but more sows performing ‘active behavior’ when having older enrichment material. However, the renovation of enrichment material was not recorded during data collection, wherefore this possible influence cannot be verified and needs to be addressed in further studies.

In contrast to the present study and the study by Czycholl et al. [30], Temple et al. [10] detected low test−retest reliability of the ISS in growing pigs for all categories when applying the reliability parameters RS and ICC and the agreement parameter LoA. Contrastingly, they explained the low test−retest reliability between the farm visits of their study by variations in the mean prevalence due to inconsistency in the observer resulting from an elevated experience of the observer during the data collection. Concerning their experimental setup, Temple et al. [10] applied the Welfare Quality^®^ protocol for growing pigs on 15 farms. Thereby, they used an assessment interval of on average twelve months. Within the present study, thirteen farms were visited five times each in the period from September 2016 to April 2018. When comparing both study designs, Temple et al. [10] executed 30 farm visits compared to the 65 farm visits of the present study and Temple et al. [10] had an inactive phase between the repetitions, whereas the data collection of the present study was performed continuously. Thus, practice and experience of the observer should be greater in the present study. At the same time, training is highly recommended to overcome biasing factors in the data collection [36]. Further, constant practice is advised [40] and the positive influence of practice on reliability has been reported [8]. Thus, the experimental setup of the present study applying training before data collection and providing continuous practice should have contributed to avoid observer drift, which adds validity to the results of the present study.

### 4.4. Human−Animal Relationship Test (HAR)

Withdrawal from humans or avoidance of humans, commonly summarized as ‘fear responses’, are part of defined behavioral patterns [41] and can be used to assess animal welfare as they are direct responses of the animals to potentially dangerous situations in their environment [42]. Fear can severely affect the welfare of animals, productivity, product quality, and finally the profitability of the farm and is therefore a major problem in animal husbandry [43]. Thus, animals’ reactions to for instance restraint are correlated to productivity and product quality [44,45] and high fearfulness is linked with reduced performance [42]. Thereby, fear of humans is widely influenced by the way the stock personnel interacts with the animals [46].

In the present study, acceptable reliability was achieved in terms of the reliability parameters RS and ICC over all farm visits for the HAR in category 0. In contrast, the consistency over time in terms of RS and ICC was low for category 1 and category 2. Further, the agreement parameters SDC and LoA indicated low agreement in all categories (i.e., the values obtained during the farm visits are not directly equivalent to each other).

With regard to other studies, the results for the test−retest reliability of the HAR in growing pigs are ambivalent. Temple et al. [10] reported good test−retest reliability for the HAR. They compared their results with studies performed in cattle [47,48].

De Passillé and Rushen [49] questioned the results of the study performed by Rousing and Waiblinger [47]. Rousing and Waiblinger [47] evaluated the test−retest reliability of an avoidance test in cattle, which showed a degree of concordance of 53.3% and a kappa coefficient of 0.37, as satisfactory. Likewise, Temple et al. [10] reported a RS of 0.69 and an ICC of 0.51. De Passillé and Rushen [49] argued that statistically significant correlation does not always go together with a large amount of variance shared by the study objects under comparison. Especially with moderate correlation, as can be seen in the results of Rousing and Waiblinger [47], a large number of misclassification can be found. Thus, de Passillé and Rushen [49] raised the concern that no thresholds exist to define acceptable reliability. Further, de Passillé and Rushen [49] disbelieved in the overall validity of the HAR. They pointed out that the HAR may be too sensitive and could be influenced by minor effects. Effects on the reliability of the HAR were accordingly supposed by Czycholl et al. [30]. In contrast to Temple et al. [10], Czycholl et al. [30] detected insufficient test−retest reliability for the HAR in growing pigs. Concerning the supposed effects, such as for instance age, Czycholl et al. [30] suggested further analyses to test for significant effects. In another study on growing pigs, Krugmann et al. [50] added the effect of habituation and concluded that the use of behavioral tests such as the HAR as single indicators to assess the animals’ affective state, which takes part in the principle ‘appropriate behavior’, is not advised.

In closing, the results of the present study found an acceptable reliability for category 0 of the HAR. The correlation could only be rated as moderate bearing in mind the concerns of de Passillé and Rushen [49]. Further, no agreement could be detected in any of the categories. Thus, the test−retest reliability of the HAR is inadequate and the HAR is not recommended to assess animal welfare over time. Concerning the suggestions of Czycholl et al. [30] and Krugmann et al. [50], future studies are needed to uncover potential effects such as age, habituation, or season on the HAR.

### 4.5. Stereotypies (ST)

Stereotypical behaviors are appreciated as indicators of animal welfare because they exhibit the response of an animal to a stressful or long-term aversive environment. Regarding sows in the gestation unit, stereotypical behaviors are commonly related to frustration due to restrictive feeding but may also be influenced by low environmental enrichment [51].

The indicators sham chewing and tongue rolling showed acceptable to good reliability concerning the reliability parameters RS and ICC, while the values achieved by the agreement parameters SDC and LoA exceeded the limits established in literature and applied throughout the manuscript. The good reliability of sham chewing signifies that it was possible to explain a high amount of variance between the farm visits under comparison [49]. Thus, the indicator can be used to differentiate between farms even though the values of the farm visits are not in agreement. On the downside, the present study certainly detected acceptable reliability for tongue rolling. However, bearing in mind the concerns of de Passillé and Rushen [49], acceptable reliability corresponds to a moderate correlation. Thus, a high amount of variance is not explained. Consequently, the consistency of tongue rolling over time is questionable. Therefore, it cannot be recommended for the assessment of animal welfare.

In contrast, the indicators teeth grinding, bar, drinker, trough biting, and floor licking achieved only poor reliability regarding the reliability parameters RS and ICC, but good agreement in terms of the agreement parameters SDC and LoA. The results can once again be explained by the influences on RS and ICC described above. Hence, the low reliability of teeth grinding, bar, drinker, trough biting, and floor licking can be explained by the low prevalence of these indicators. Thus, the test−retest reliability of these indicators can be rated as acceptable.

In sum, the test−retest reliability of ST can be rated as acceptable—with doubts for the indicator tongue rolling.

## 5. Conclusions

The present study aimed at testing the ‘Welfare Quality^®^ animal welfare assessment protocol for sows and piglets’ in respect to its test−retest reliability in an on-farm study on breeding to wean production animals. The present paper focused on the evaluation of the assessment of the welfare principle of ‘appropriate behavior’. The results with regard to the remaining welfare principles (‘good feeding’, ‘good housing’ and ‘good health’) are published in the second part of the study. As a result of the evaluation of the test−retest reliability of the principle ‘appropriate behavior’, the QBA achieved poor test−retest reliability. Acceptable test−retest reliability could be detected for most parts of the ISS but not for the categories ‘use of enrichment material’ and ‘other active behavior’. The HAR achieved poor test−retest reliability in total. Acceptable test−retest reliability could be found for ST. In conclusion, the assessment of the welfare principle of ‘appropriate behavior’ of the ‘Welfare Quality^®^ animal welfare assessment protocol for sows and piglets’ lacks consistency over time to accurately assess animal welfare in sows. Adaptions to the QBA are necessary to find an unambiguous definition for the adjectives. The influence of certain effects as renovation of enrichment material, age, habituation, or season on certain categories of the ISS and the HAR in general needs to be further investigated. In conclusion, this study is one of the first studies to examine the test−retest reliability of the Welfare Quality^®^ protocol for sows and piglets; with its results, this study is capable of having an impact on the improvement of the protocol.

## Figures and Tables

**Table 1:** Overview of the thirteen farms, which took part in the present study. Presented are: production type, herd size, and production rhythm (weeks).

Farm	Production Type	Herd Size	Production Rhythm, Weeks
1	Conventional	400	1
2	Conventional	120	3
3	Conventional	330	1
4	Conventional	5000	1
5	Conventional	150	2
6	Conventional	80	3
7	Organic	40	3
8	Conventional	810	1
9	Conventional	180	1
10	Conventional	240	3
11	Conventional	330	1
12	Conventional	1000	4
13	Organic	50	3

**Table 2:** Mean values (%) and standard errors (SE) of the five farm visits (1–5) for the adjectives of the Qualitative Behavior Assessment.

Adjectives	Farm Visit, % (SE)				
	1	2	3	4	5
Active	36.7 (7.53)	64.4 (6.92)	47.0 (7.83)	55.7 (5.15)	45.2 (5.51)
Relaxed	79.3 (6.14)	69.3 (7.92)	66.3 (8.32)	73.5 (6.01)	72.9 (5.40)
Fearful	4.74 (1.25)	7.45 (3.34)	9.91 (3.58)	11.4 (4.93)	6.95 (1.87)
Agitated	16.1 (5.25)	27.6 (7.62)	30.2 (7.39)	22.7 (5.00)	21.2 (4.44)
Calm	85.1 (5.60)	72.3 (8.04)	70.7 (8.85)	80.9 (5.11)	77.4 (5.12)
Content	64.1 (6.27)	62.3 (5.53)	54.8 (6.22)	56.1 (5.94)	50.2 (5.49)
Tense	11.3 (5.78)	21.1 (6.59)	25.2 (6.48)	19.3 (4.72)	24.3 (4.48)
Enjoying	54.8 (7.17)	55.5 (7.46)	49.7 (6.39)	52.3 (5.94)	47.9 (4.33)
Frustrated	15.6 (5.98)	17.9 (4.71)	23.6 (6.05)	31.1 (5.03)	33.1 (5.28)
Sociable	54.3 (6.76)	58.6 (6.31)	58.9 (5.31)	63.1 (4.40)	57.8 (3.67)
Bored	24.6 (7.67)	15.3 (2.95)	30.2 (6.89)	36.6 (6.97)	48.9 (7.08)
Playful	15.4 (4.40)	9.66(3.17)	20.7 (5.31)	27.6 (4.98)	35.8 (5.15)
Positively occupied	59.3 (8.42)	65.0 (6.60)	53.2 (8.32)	53.2 (5.79)	47.6 (5.63)
Listless	9.42 (4.84)	4.55 (1.15)	8.55 (2.12)	16.1 (3.58)	21.2 (3.86)
Lively	16.8 (6.49)	20.1 (7.72)	20.2 (6.67)	16.4 (5.26)	23.0 (6.61)
Indifferent	69.8 (7.32)	66.0 (7.62)	69.2 (6.93)	78.2 (4.94)	76.3 (6.85)
Irritable	6.89 (3.57)	8.49 (3.08)	8.68 (3.71)	11.0 (3.17)	9.17 (2.06)
Aimless	12.6 (5.01)	12.7 (3.18)	20.4 (6.14)	26.8 (5.39)	28.3 (4.24)
Happy	69.6 (5.66)	61.7 (5.75)	53.1 (6.24)	50.7 (5.70)	49.4 (4.54)
Distressed	3.57 (2.03)	3.88 (1.53)	13.5 (4.54)	13.2 (2.84)	17.2 (3.91)

**Table 3:** Statistical parameters (Spearman’s rank correlation coefficient: RS, intraclass correlation coefficient: ICC, smallest detectable change: SDC, limits of agreement: LoA) for the comparison of the five farm visits (1–5) for the adjectives of the Qualitative Behavior Assessment, indicating poor (normal type, gray color), acceptable (normal type, black color), and good (bold type, black color) reliability or agreement, respectively.

Adjectives	RS				ICC				SDC				LoA			
	1–2	1–3	1–4	1–5	1–2	1–3	1–4	1–5	1–2	1–3	1–4	1–5	1–2	1–3	1–4	1–5
Active	0.30	**0.73**	−0.35	0.06	0.40	**0.76**	0.00	0.31	0.62	0.40	0.70	0.54	−0.83 to 0.28	−0.48 to 0.27	−0.91 to 0.53	−0.63 to 0.46
Relaxed	0.34	**0.63**	0.22	0.36	0.40	**0.77**	0.17	0.23	0.55	0.40	0.55	0.51	−0.45 to 0.65	−0.22 to 0.48	−0.50 to 0.61	−0.44 to 0.57
Fearful	0.45	0.59	−0.32	−0.06	0.42	0.29	0.00	0.00	0.17	0.21	0.33	0.16	−0.22 to 0.16	−0.28 to 0.17	−0.45 to 0.31	−0.19 to 0.14
Agitated	**0.73**	**0.83**	0.06	0.36	0.56	**0.73**	0.25	0.33	0.45	0.39	0.44	0.40	−0.55 to 0.32	−0.47 to 0.19	−0.51 to 0.38	−0.45 to 0.34
Calm	**0.77**	**0.77**	0.28	0.23	0.68	**0.80**	0.36	0.49	0.42	0.39	0.43	0.38	−0.26 to 0.52	−0.19 to 0.47	−0.39 to 0.47	−0.31 to 0.46
Content	0.37	0.55	**0.74**	0.28	0.19	0.65	**0.74**	0.36	0.53	0.39	0.31	0.47	−0.51 to 0.55	−0.27 to 0.46	−0.23 to 0.39	−0.33 to 0.61
Tense	0.49	0.60	0.24	−0.03	0.28	0.20	0.22	0.40	0.53	0.56	0.46	0.40	−0.62 to 0.43	−0.69 to 0.41	−0.55 to 0.38	−0.53 to 0.27
Enjoying	0.31	0.69	**0.75**	0.51	0.42	0.68	0.66	0.49	0.55	0.38	0.38	0.41	−0.56 to 0.55	−0.33 to 0.43	−0.36 to 0.41	−0.35 to 0.49
Frustrated	0.33	0.65	0.57	0.25	0.53	**0.78**	0.47	0.17	0.36	0.30	0.40	0.51	−0.39 to 0.35	−0.36 to 0.20	−0.55 to 0.25	−0.69 to 0.34
Sociable	**0.93**	**0.70**	0.40	0.29	**0.87**	0.69	0.28	0.18	0.24	0.34	0.47	0.47	−0.28 to 0.19	−0.39 to 0.30	−0.57 to 0.40	−0.53 to 0.46
Bored	0.38	0.34	0.61	0.19	0.36	**0.89**	**0.85**	0.34	0.43	0.26	0.28	0.60	−0.37 to 0.56	−0.30 to 0.19	−0.40 to 0.16	−0.84 to 0.36
Playful	0.07	**0.73**	0.08	0.53	0.00	0.59	0.00	0.29	0.40	0.31	0.50	0.40	−0.35 to 0.46	−0.37 to 0.26	−0.62 to 0.38	−0.61 to 0.20
Positively occupied	0.29	0.65	**0.84**	0.27	0.22	0.56	**0.77**	0.29	0.66	0.50	0.34	0.59	−0.73 to 0.61	−0.44 to 0.57	−0.28 to 0.40	−0.49 to 0.72
Listless	0.46	0.29	0.14	0.23	0.33	0.29	0.37	0.19	0.25	0.29	0.33	0.39	−0.24 to 0.34	−0.30 to 0.32	−0.40 to 0.27	−0.51 to 0.27
Lively	**0.74**	**0.73**	0.20	0.54	**0.79**	**0.91**	0.21	0.51	0.32	0.20	0.52	0.46	−0.36 to 0.30	−0.23 to 0.16	−0.52 to 0.53	−0.52 to 0.40
Indifferent	0.11	−0.20	−0.55	0.25	0.39	0.00	0.00	0.18	0.58	0.74	0.75	0.64	−0.55 to 0.62	−0.73 to 0.74	−0.85 to 0.68	−0.71 to 0.57
Irritable	0.20	0.41	−0.09	0.30	**0.87**	**0.89**	0.00	0.40	0.12	0.12	0.34	0.22	−0.13 to 0.10	−0.14 to 0.10	−0.38 to 0.30	−0.25 to 0.20
Aimless	0.16	0.33	0.42	0.28	0.26	0.44	0.63	0.39	0.35	0.42	0.32	0.36	−0.36 to 0.36	−0.50 to 0.34	−0.46 to 0.17	−0.52 to 0.21
Happy	0.60	0.65	**0.72**	0.60	0.58	**0.81**	**0.70**	0.54	0.38	0.36	0.31	0.34	−0.29 to 0.45	−0.10 to 0.43	−0.12 to 0.50	−0.14 to 0.55
Distressed	0.50	0.50	0.18	0.20	0.62	0.26	0.14	0.10	0.11	0.29	0.23	0.28	−0.11 to 0.11	−0.40 to 0.20	−0.33 to 0.13	−0.43 to 0.16

**Table 4:** Spearman’s rank correlation coefficient (RS) for the comparison of the factor loadings on the first two principal components (PC; PC1 and PC2) between farm visits.

Comparison	RS (PC1)	RS (PC2)
Farm visit 1 to farm visit 2	−0.83	−0.80
Farm visit 1 to farm visit 3	0.93	0.87
Farm visit 1 to farm visit 4	0.93	−0.12
Farm visit 1 to farm visit 5	−0.90	0.83

**Table 5:** Spearman’s rank correlation coefficient (RS) for the comparison of the factor loadings on the first two principal components (PC; PC1 and PC2) between farm visits for the groups^1^ determined by correlation between the adjectives of the Qualitative Behavior Assessment.

Group	Comparison	RS (PC1)	RS (PC2)
1	Farm visit 1 to farm visit 2	−0.94	−0.14
	Farm visit 1 to farm visit 3	0.71	0.37
	Farm visit 1 to farm visit 4	0.77	0.43
	Farm visit 1 to farm visit 5	−0.37	0.71
2	Farm visit 1 to farm visit 2	−0.82	0.53
	Farm visit 1 to farm visit 3	0.93	0.82
	Farm visit 1 to farm visit 4	0.93	0.68
	Farm visit 1 to farm visit 5	−0.89	0.57
3	Farm visit 1 to farm visit 2	−0.64	−0.32
	Farm visit 1 to farm visit 3	0.64	0.11
	Farm visit 1 to farm visit 4	0.68	−0.79
	Farm visit 1 to farm visit 5	−0.42	0.21

^1^ Group 1 includes the adjectives ‘relaxed’, ‘happy’, ‘enjoying’, ‘content’, ‘positively occupied’, ‘sociable’; Group 2 includes the adjectives ‘lively’, ‘playful’, ‘irritable’, ‘active’, ‘fearful’, ‘agitated’, ‘tense’; Group 3 includes the adjectives ‘aimless’, ‘bored’, ‘frustrated’, ‘distressed’, ‘listless’, ‘calm’, ‘indifferent’.

**Table 6:** Mean values (%) and standard errors (SE) of the five farm visits (1–5) for the categories of the behavioral observations by instantaneous scan sampling.

Category	Farm Visit, % (SE)				
	1	2	3	4	5
Positive social behavior	1.82 (0.80)	1.62 (0.65)	0.88 (0.34)	0.65 (0.29)	1.30 (0.42)
Negative social behavior	0.54 (0.25)	1.20 (0.42)	1.03 (0.29)	0.62 (0.29)	0.88 (0.37)
Use of enrichment material	18.3 (6.16)	20.3 (6.27)	18.5 (7.49)	18.5 (6.40)	13.0 (5.15)
Investigation of the pen	1.82 (0.80)	1.62 (0.65)	0.88 (0.34)	0.65 (0.29)	1.30 (0.42)
Other active behavior	77.5 (5.89)	75.3 (6.36)	78.8 (7.24)	79.6 (6.27)	83.6 (4.94)

**Table 7:** Statistical parameters (Spearman’s rank correlation coefficient: RS, intraclass correlation coefficient: ICC, smallest detectable change: SDC, limits of agreement: LoA) for the comparison of the five farm visits (1–5) for the categories of the behavioral observations by instantaneous scan sampling, indicating poor (normal type, gray color), acceptable (normal type, black color), and good (bold type, black color) reliability or agreement, respectively.

Category	RS				ICC				SDC				LoA			
	1–2	1–3	1–4	1–5	1–2	1–3	1–4	1–5	1–2	1–3	1–4	1–5	1–2	1–3	1–4	1–5
Positive social behavior	0.07	0.47	**0.72**	0.26	0.00	0.16	0.45	0.40	0.08	**0.05**	**0.04**	**0.05**	−0.08 to 0.08	−0.05 to 0.07	**−0.03 to 0.06**	**−0.04 to 0.05**
Negative social behavior	0.24	0.45	0.23	0.44	0.05	0.37	0.19	0.11	**0.03**	**0.02**	**0.02**	**0.03**	**−0.04 to 0.03**	**−0.03 to 0.02**	**−0.03 to 0.02**	**−0.03 to 0.03**
Use of enrichment material	**0.73**	**0.85**	**0.83**	0.47	0.61	**0.86**	0.64	0.32	0.39	0.25	0.38	0.46	−0.41 to 0.37	−0.26 to 0.25	−0.38 to 0.38	−0.41 to 0.52
Investigation of the pen	0.07	0.47	**0.72**	0.26	0.00	0.16	0.45	0.40	0.08	**0.05**	**0.04**	**0.05**	−0.08 to 0.08	−0.05 to 0.07	**−0.03 to 0.06**	**−0.04 to 0.05**
Other active behavior	0.55	**0.72**	**0.82**	0.20	0.57	**0.84**	0.60	0.26	0.40	0.26	0.38	0.46	−0.38 to 0.42	−0.28 to 0.25	−0.40 to 0.36	−0.53 to 0.40

**Table 8:** Mean values (%) and standard errors (SE) of the five farm visits (1–5) for the categories of the human−animal relationship test and the categories of the assessment of stereotypies.

Indicator	Category	Farm Visit, % (SE)				
		1	2	3	4	5
Human-animal relationship test	0	44.1 (5.93)	52.8 (5.53)	59.8 (6.43)	65.3 (7.05)	55.1 (6.77)
	1	17.0 (3.72)	15.1 (3.12)	15.3 (3.38)	14.9 (4.77)	23.0 (5.10)
	2	38.9 (7.38)	32.1 (4.99)	24.9 (6.40)	19.8 (5.71)	21.9 (7.90)
Stereotypies						
Sham chewing	2	19.5 (5.03)	20.9 (5.24)	15.6 (3.67)	21.4 (5.23)	25.8 (5.84)
Tongue rolling	2	7.13 (3.35)	7.36 (2.31)	5.19 (1.69)	4.83 (1.61)	6.76 (1.51)
Teeth grinding	2	0.19 (0.19)	1.25 (0.69)	0.77 (0.44)	0.38 (0.26)	0.19 (0.19)
Bar, drinker, trough biting	2	0.59 (0.42)	1.54 (1.15)	1.15 (0.61)	0.58 (0.30)	1.65 (0.90)
Floor licking	2	0.58 (0.42)	1.35 (0.67)	1.35 (0.61)	2.50 (1.10)	2.93 (0.85)

**Table 9:** Statistical parameters (Spearman’s rank correlation coefficient: RS, intraclass correlation coefficient: ICC, smallest detectable change: SDC, limits of agreement: LoA) for the comparison of the five farm visits (1–5) for the categories of the human−animal relationship test and the categories of the assessment of stereotypies, indicating poor (normal type, gray color), acceptable (normal type, black color), and good (bold type, black color) reliability or agreement, respectively.

Indicator	Category	RS				ICC				SDC						LoA	
		1–2	1–3	1–4	1–5	1–2	1–3	1–4	1–5	1–2	1–3	1–4	1–5	1–2	1–3	1–4	1–5
Human animal relationship test	0	**0.73**	0.42	0.41	0.53	**0.73**	0.46	0.51	0.53	0.30	0.45	0.46	0.44	−0.38 to 0.21	−0.61 to 0.30	−0.67 to 0.25	−0.55 to 0.33
	1	0.62	0.43	0.26	0.58	**0.75**	0.28	0.06	0.35	0.17	0.30	0.41	0.36	−0.15 to 0.19	−0.29 to 0.32	−0.39 to 0.44	−0.42 to 0.30
	2	**0.71**	0.52	0.63	0.24	0.69	0.69	0.69	0.47	0.35	0.38	0.36	0.56	−0.28 to 0.42	−0.24 to 0.52	−0.18 to 0.56	−0.39 to 0.73
Stereotypies																	
Sham chewing	0/2	**0.81**	**0.93**	**0.83**	**0.85**	**0.80**	**0.85**	**0.91**	**0.91**	0.23	0.17	0.15	0.16	−0.24 to 0.21	−0.13 to 0.21	−0.17 to 0.13	−0.22 to 0.10
Tongue rolling	0/2	**0.73**	0.69	0.62	0.57	0.68	0.62	0.61	0.43	0.16	0.15	0.16	0.18	−0.17 to 0.16	−0.14 to 0.18	−0.14 to 0.19	−0.19 to 0.20
Teeth grinding	0/2	−0.19	−0.16	−0.12	−0.08	0.00	0.00	0.00	0.00	**0.05**	**0.03**	**0.02**	**0.02**	**−0.06 to 0.04**	**−0.04 to 0.03**	**−0.03 to 0.02**	**−0.02 to 0.02**
Bar, drinker, trough biting	0/2	0.39	0.24	0.31	0.48	0.54	0.27	0.38	0.09	**0.05**	**0.04**	**0.03**	0.06	−0.07 to 0.05	**−0.05 to 0.04**	**−0.03 to 0.03**	−0.08 to 0.06
Floor licking	0/2	0.32	−0.33	0.63	0.55	0.56	0.00	0.52	0.58	**0.04**	0.06	**0.05**	**0.04**	**−0.04 to 0.03**	−0.07 to 0.05	−0.08 to 0.04	**−0.07 to 0.02**
